# Cavum vergae in a patient with autism spectrum disorder

**DOI:** 10.1192/j.eurpsy.2025.1103

**Published:** 2025-08-26

**Authors:** C. Azizagaoglu, E. Dogan Elmas, F. O. Ciftci, O. Gulesen, F. Mavi, S. Bodur

**Affiliations:** 1 Child and Adolescent Psychiatry, Gülhane Training and Research Hospital, Ankara; 2 Child and Adolescent Psychiatry, Kirikkale Yuksek Ihtisas Hospital, Kirikkale, Türkiye

## Abstract

**Introduction:**

A neurodevelopmental disorder known as autism spectrum disorder (ASD) affects approximately 1 in 54 children. It has a complex etiology, but little is known about its neurological and genetic basis. (Loomba *et al.,* 2021 Psychiatry Research:Neuroimaging, *313*, 111301.) The septum pellucidum is a part of the limbic system, which consists of two layers of gray and white matter and forms the medial walls of the lateral ventricles. A cavum septum pellucidum (CSP) is a septum pellucidum that has a division between its two layers. All fetuses have CSP, although around 85% fuse within three to six months after birth. The posterior expansion of the CSP is called the cavum vergae (CV). Up to 30% of neonates have this structure, although less than 1% of people have it in adulthood. (Landin-Romero *et al*., 2015 Journal of Affective Disorders,186,53-57.)

**Objectives:**

The aim of this study is to understand the relationship between ASD and CV.

**Methods:**

The patient, seven year old, applied to clinic accompanied by his parents due to speech delay in January 2024. He was product of full-term delivery, born by caesarian section secondary to servical stenosis. His mother had servical bleeding in the 6th week of pregnancy which she had taken progesterone for several days.

**Results:**

During our examination, he did not respond to his name every time, maintained eye contact for a short time, did not initiate communication most of the time, did not continue even if he initiated it, showed more interest in objects than people, had echolalia and dysprosody. Based on our evaluation, we diagnosed as ASD and referred for special education. Although most of his tests were normal, CV was reported in brain MRIs (Image1). Retaining the CSP or CV after birth may indicate abnormalities in the development of the brain. Co-occurrence of enlarged CSP and CV persistent into adulthood is less common, but it has also been associated with neurodevelopmental disorders, decreased mental status, psychosis and bipolar disorder. (Landin-Romero *et al.,*2015 Journal of Affective Disorders,186,53-57.) This case, based on the idea that CV is seen more frequently in psychiatric diseases, supports the idea that neuroimaging findings that have emerged in recent years are changes in the neuroanatomical structure and brain connection of ASD.

**Image 1:**

**Figure:**
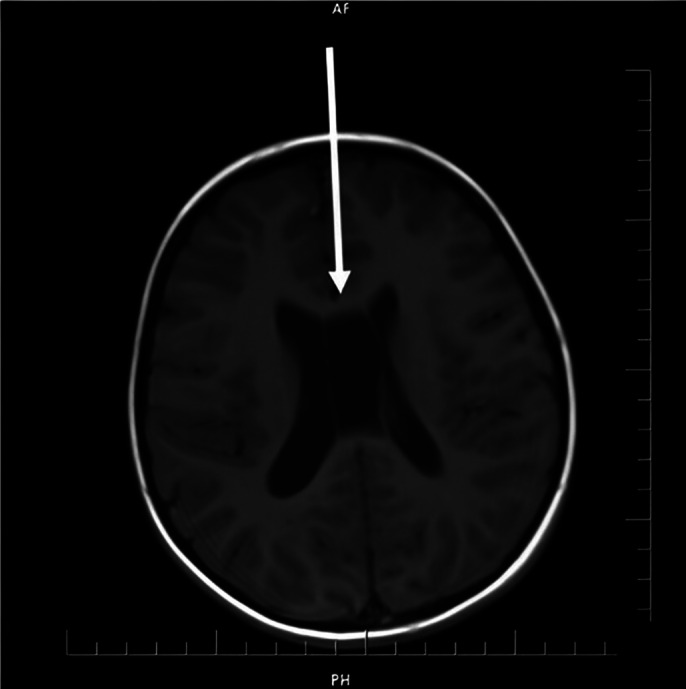

**Conclusions:**

Retaining the CSP or CV after birth may indicate abnormalities in the development of the brain. Isolated CV is rather unusual. Co-occurrence of enlarged CSP and CV persistent into adulthood is less common, but it has also been associated with neurodevelopmental disorders, decreased mental status, psychosis and bipolar disorder. (Landin-Romero *et al.,* 2015 Journal of Affective Disorders, 186, 53-57.) This case, based on the idea that CV is seen more frequently in psychiatric diseases, supports the idea that neuroimaging findings that have emerged in recent years are changes in the neuroanatomical structure and brain connection of ASD.

**Disclosure of Interest:**

None Declared

